# A 3D-Printed Do-It-Yourself ELISA Plate Reader as a Biosensor Tested on TNFα Assay

**DOI:** 10.3390/bios14070331

**Published:** 2024-07-06

**Authors:** Miroslav Pohanka, Ondřej Keresteš, Jitka Žáková

**Affiliations:** Military Faculty of Medicine, University of Defence, Trebesska 1575, CZ-50001 Hradec Kralove, Czech Republic; ondrej.kerestes@unob.cz (O.K.);

**Keywords:** biosensor, colorimetry, cytokine, ELISA, enzyme-linked immunosorbent assay, image, immunosensor, inflammation

## Abstract

Simple analytical devices suitable for the analysis of various biochemical and immunechemical markers are highly desirable and can provide laboratory diagnoses outside standard hospitals. This study focuses on constructing an easily reproducible do-it-yourself ELISA plate reader biosensor device, assembled from generally available and inexpensive parts. The colorimetric biosensor was based on standard 96-well microplates, 3D-printed parts, and a smartphone camera as a detector was utilized here as a tool to replace the ELISA method, and its function was illustrated in the assay of TNFα as a model immunochemical marker. The assay provided a limit of detection of 19 pg/mL when the B channel of the RGB color model was used for calibration. The assay was well correlated with the ELISA method, and no significant matrix effect was observed for standard biological samples or interference of proteins expected in a sample. The results of this study will inform the development of simple analytical devices easily reproducible by 3D printing and found on generally available electronics.

## 1. Introduction

Immunoassays are standard analytical methods suitable for the detection of several analytes, and are based on various physical and chemical principles, including spectrophotometry, colorimetry, electrochemistry, and radioassay. Methods such as enzyme-linked immunosorbent assay (ELISA) [1], agglutination tests [2,3], lateral flow immunochromatographic assays (also known as lateral flow tests) [4,5,6], radioimmunoassay [7,8], and biosensors with antibodies as recognition elements, also known as immunosensors [9,10,11], are the most notable. Immunoassays have broad uses. Although some immunoassays are typical laboratory methods including the ELISA method, the other, simpler methods can be performed under field conditions or as point-of-care tests (e.g., agglutination tests in tubes, and lateral flow tests). However, simple methods like agglutination tests or lateral flow tests have limitations, such as lack of quantification of the marker. 

The ELISA method was introduced in the 1970s, and the term enzyme immunoassay, or EIA, was also common at this time. ELISA gained high popularity and partially replaced radioimmunoassay because no substantially hazardous material is needed for ELISA. The ELISA method has some further substantial advantages, making it a widespread tool in various laboratories, including healthcare facilities, environmental laboratories, and the food industry, because of its relatively high sensitivity and suitability even for markers with low concentrations, and the ability to measure multiple samples simultaneously. Standard ELISA uses microplates with 96 wells, but other arrangements (384 or 1536 wells) are also possible. The principle of the methods also has an advantage in the multiplication of signals. Antibodies labeled directly or indirectly interact with the analyte. One analyte molecule rises to a much higher number of colored reagents as they are gradually converted by the enzyme linked to the antibody. The coloration can be further improved by prolonging the incubation time when the analyte is present at low concentration. 

Photogrammetry has recently been used in some works as a platform for immunoassays. Analysis of color depth in each well using red, green, and blue channels is an emerging technique in point-of-care testing methods. However, a simple recording of a well plate is problematic, due to differences in light conditions potentially occurring in each well. This problem was addressed from different perspectives in the works of Bergua et al. and Volpe et al. [12,13]. Although these portable instruments offer smartphone connectivity, they need a microcontroller platform and thorough programming for proper operation. On the other hand, there is work from Bagheri et al. in which the proposed platform consists of a wax-printed 96-well plate ready for colorimetry [14]. Smartphones were also used in the immunoassays for the detection of meat adulteration [15], carcinoembryonic antigen assay [16], and pairing with lateral flow tests [17]. 

The assay of tumor necrosis alpha (TNFα) was chosen as the biosensor testing method because TNFα is a common immunochemical marker that occurs during inflammation and various diseases, with the involvement of the innate immune response [18,19,20]. TNFα occurs in the body fluids in quite low concentrations, making it a relatively hard marker to analyze by simple point-of-care tests. Concentrations in the range of pg/mL can be expected, for example, in the blood plasma [21,22,23].

One of the current trends in the construction of immunoassays and analytical means from a global perspective is focused on the miniaturization and simplification of the devices to make them affordable and easy to operate. Newly invented devices can be designed in this way. Biosensors specific to TNFα are an alternative to the standard immunoassays. An electrochemical biosensor comprising metal–organic frameworks, and a modified gold electrode with monoclonal antibodies was used for the assay TNFα with a limit of detection of 0.15 pg/mL [24]. In another study, a lab-on-a-chip calorimetric biosensor was constructed to measure TNFα in the astrocyte’s cell culture media with a limit of detection of 14 pg/mL [25]. A relatively low limit of detection of 2 fg/mL was achieved by an assay of a voltametric immunosensor working with gold nanoparticles modified with multi-walled carbon nanotubes and a bimetallic sensor [26]. The newly constructed biosensors are quite effective analytical devices. Still, they can also have some drawbacks, including problems with mass production due to new technologies not common in the industry, limited possibility to rebuild them for other analytes, and high prices for the used advanced materials and technologies.

**Figure 1:**
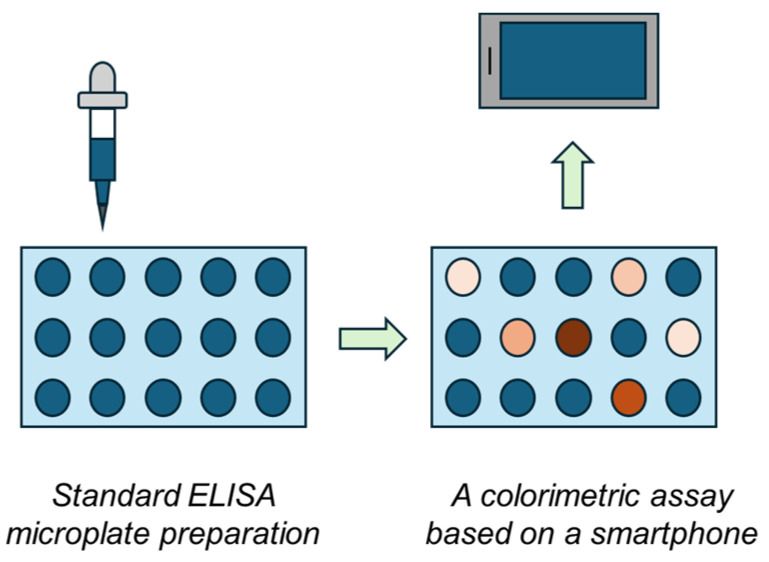
The general principle of the colorimetric biosensor based on a smartphone and ELISA microplates.

The ELISA method is a standard method where significant changes in the assay principle are possible because universality should be maintained; however, simplification would make the assay more accessible outside laboratories. A microplate reader is a device necessary for the ELISA method and it is also a relatively expensive spectrophotometer, as multiple channels need to be processed simultaneously. In recent years, new approaches were introduced to make ELISA more available, and though they are not ready for immediate application, they provide a functional outcome [27,28,29,30]. This study focuses on constructing an easily reproducible do-it-yourself ELISA plate reader biosensor device assembled from generally available and inexpensive parts. It contains a common smartphone camera and a 3D-printed box that provides constant illumination. This biosensor device is considered a cheap alternative to microplate-based assays, allowing the ELISA to be performed even outside standard laboratories. The general principle of the idea is depicted in Figure 1.

## 2. Materials and Methods

### 2.1. ELISA for TNFα

ELISA was performed on 96-well microplates with flat well bottoms of PolySorp type (Thermo Fisher Scientific, Waltham, MA, USA). TNFα of human type (Sigma-Aldrich, Merck Group, St. Louis, MO, USA) solved in phosphate-buffered saline with a pH of 7.4 (PBS), or spiked in human volunteer blood plasma, PBS spiked with various proteins, or pure PBS served as samples. 

A sample with a volume of 100 µL was applied per well, and microplates were covered with a self-adhesive lid and incubated at room temperature under orbital shaking. Subsequently, the solution was poured, and the wells were washed four times (200 µL per cycle and well) by PBS with 0.05% Tween 20. The washed wells were blocked with 100 μL of 0.1% gelatin, covered with a self-adhesive lid and placed in an orbitally shaken box with a temperature set at 37 °C for one hour, and then washed again four times with PBS with Tween 20. In the next step, 100 µL of an anti-human TNFα antibody of IgG type produced in rabbits (Sigma-Aldrich) diluted 1:100 was injected per well, and the plates were covered by a self-adhesive lid and incubated at 37 °C with constant orbital shaking for 60 min. After repeated washing with PBS with 0.05% (*v*/*v*) Tween 20, an anti-rabbit IgG polyclonal antibody labeled with horseradish peroxidase and produced in goats (Sigma-Aldrich) was dissolved 1:100 in PBS and applied in an amount of 100 µL per well; the microplates were covered with a self-adhesive lid and subjected to incubation at 37 °C with constant orbital shaking for 60 min, and the wells were washed with PBS with Tween 20. Then, 100 μL of a fresh solution containing a liquid substrate 3,3′,5,5′-tetramethylbenzidine for ELISA (Sigma-Aldrich) was added and left to incubate for 40 min. The reaction was stopped by adding 1 mol/L of sulfuric acid in an amount of 100 μL and optical density was measured by ELISA reader MRX (Dynatech Laboratories, Chantilly, VA, USA) at 450 nm or by the newly developed biosensor. The blank assay was based on the analysis of PBS processed as a sample. 

### 2.2. The 3D-Printed Colorimetric Biosensor and Its Use

The 3D models were printed on a Prusa i3 MK3S+ (Prusa Research, Prague, Czech Republic) printer using white acrylonitrile styrene acrylate (ASA) and polyethylene terephthalate glycol (PETG) filament (Prusa Research), with a diameter of 1.75 mm. PETG was used only for the mobile phone adapter, while the other parts had to be printed by ASA because the light source used is powerful and emits heat that would degrade the PETG-printed cells with prolonged use of the device. 

The parts were printed at 20% fill (gyroid mode) with a minimum of 3 perimeters. The filament temperature was standard −230 °C for PETG, 60 °C pad; 250 °C for ASA, 110 °C pad. 

**Figure 2:**
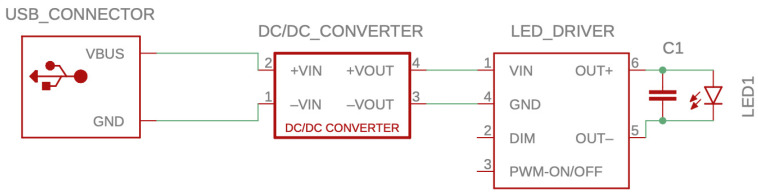
Circuit diagram for LED module used in the biosensor. The number of contacts is indicated in the diagram.

The reflector parts were bonded using an acetone ASA solution (0.05 g/mL). A smaller part, containing the heat sink-indicated LED module, was inserted from the inside into the shade. An array of four LEDs (red 2.25–2.6 V DC 620–630 nm, green 3.3–2.9 V DC 520–535 nm, blue 3.1–3.7 V DC 450–465 nm, white 3.1–3.7 V DC 5700–8000 K) total power 12 W, light angle 120°, and luminous flux 370–430 lm (Cree 12W XML RGBW, CreeLED, Inc., 4001 E Hwy 54, Suite 2000, Durham, NC 27709 USA). The circuit diagram of the LED module is shown in Figure 2.

After the reflector was glued together, a milky Plexiglas (128 × 85 mm, 67% transmittance), wrapped on two sides with an office rubber band was inserted. Next, the LED module with a heat sink was attached to the prepared projections with binding wire. 

The power cable was then fed through a hole in the base and connected to the rest of the electronics.

**Figure 3:**
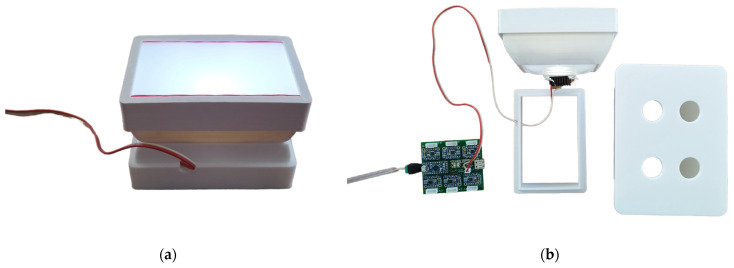

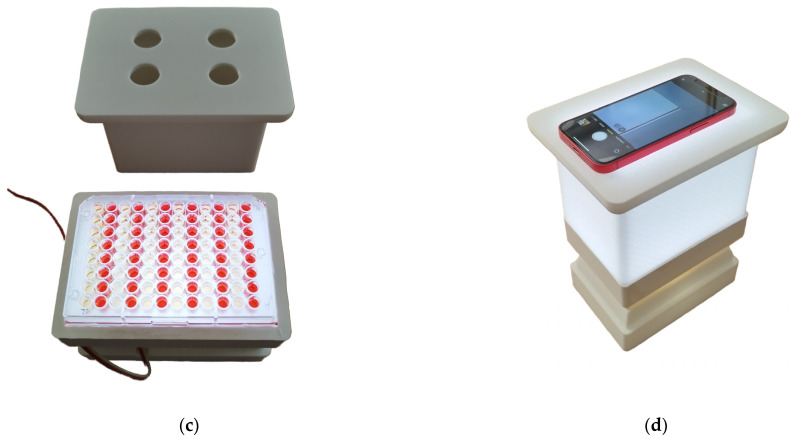
Illustrative photographs of 3D-printed colorimetric biosensors. Photographs: (**a**) the LED reflector; (**b**) the whole device disassembled; (**c**) the LED reflector with a microplate and smartphone platform; (**d**) the whole device assembled with a smartphone.

A smartphone (Redmi Note 11 Pro, Xiaomi, Haidian District, Beijing, China) was placed on the mobile phone adapter and used as a detector for coloration. The same microplates as those used for ELISA (see the previous section) were assayed by the biosensor. The standard camera mode was selected, while the integrated camera flash was switched off. Illustrative photographs of the entire device are depicted in Figure 3.

### 2.3. Data Processing

The same microplates were measured by the ELISA reader and the newly constructed biosensor. Every sample was measured in five repeats by both ELISA and the biosensor assay. The 8-bit photographs in jpg format were taken by the smartphone-integrated camera and were processed for color depth at five randomly selected points, taken from the area of half of the semi-diameter. One photograph simultaneously covered 24 wells of a microplate. A color depth value was determined for each point, and each color channel of the RGB color model using the free and open-source GIMP 2.10.36. Finally, the difference in color depth was calculated from the average color depths of the sample and the blank assay using the following equation: Δ Color depth = Color depth (blank) − Color depth (sample). The limit of detection was calculated as the point on the calibration curve equal to the triplicate of the control assay (rule signal to noise equal to three). Statistical significance was tested for parametric ANOVA for probability levels *p* = 0.05 and *p* = 0.01.

## 3. Results and Discussion

**Figure 4:**
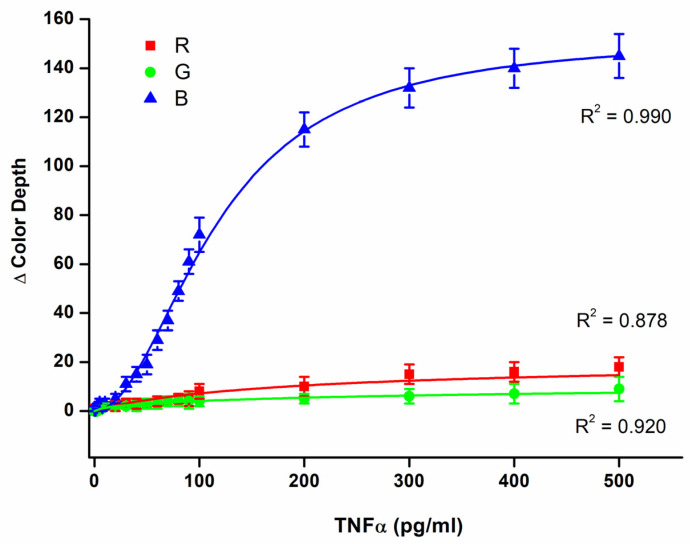
Assay of TNFα solved in PBS using a 3D-printed colorimetric biosensor and a standard 96-well microplate. Color depths for each RGB color channel were determined and calibration curves for each color channel were made. Error bars indicate the standard deviation for five repeats.

The biosensor was calibrated for TNFα solved in PBS using the range of mass 0, 1.0, 2.5, 5.0, 7.5, 10, 20, 30, 40, 50, 60, 70, 80, 90, 100, 200, 300, 400, and 500 pg/mL in a 100 µL sample. The calibration for the three-color channels is depicted in Figure 4. Of the three channels, the B channel had the best sensitivity. The dynamic range for the color depth was equal to 144, and the limit of detection was calculated to be 19 pg/mL when the B channel served as the assay. The R and G channels provided significantly worse results. The dynamic range for the color depth was equal to 14 for the R channel and 8 for the G channel. The limit of detection reached 44 pg/mL for the R channel and 56 pg/mL for the G channel. Because the B channel provided the best sensitivity, it was chosen for further experiments. The best limit of detection can cover potential pathologies in which inflammation takes place and TNFα serves as a marker. For example, serious pathologies like sepsis can cause an increase in TNFα blood levels to the average level of around 700 pg/mL [31]. On the other hand, the healthy population has a TNFα blood level of around 8 pg/mL [32], which is less than the limit of detection of the biosensor presented here. For comparison, the standard method ELISA used in this work as a reference method reached the limit of detection of 2.1 pg/mL, which provides an advantage over the biosensor assay, and more effectively covers concentrations of TNFα blood levels in healthy individuals. Despite the lower sensitivity of the biosensor assay compared to the ELISA, the biosensor assay is fully applicable to cover inflammation, and can be used to detect TNFα in patients suffering from pathologies where TNFα serves as a marker and to distinguish them from healthy individuals. The budget smartphone camera and light source are responsible for the lower sensitivity of the biosensor assay compared to the standard method ELISA. The camera chip, with a size of 1/1.52”, a pixel size of 0.7 µm, and 8-bit output with a dynamic range of color depth 0–255, are of course the main limiting factors. 

**Figure 5:**
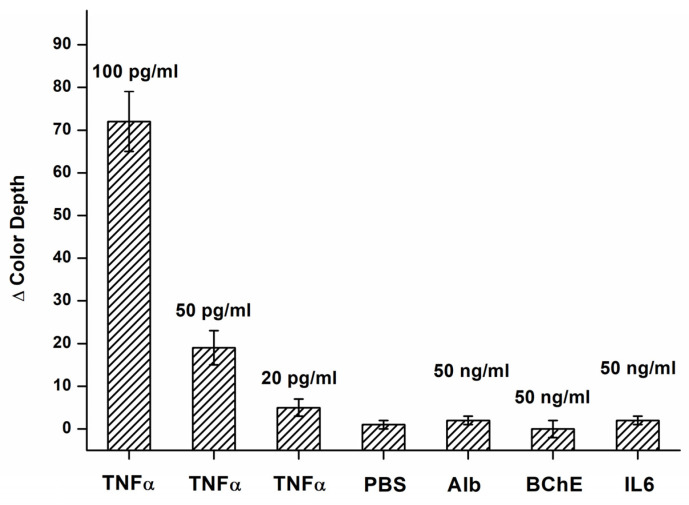
Interference tests for an assay by the colorimetric biosensor in the B channel. Results for various concentrations of TNFα, and solutions of albumin (Alb), butyrylcholinesterase (BChE), interleukin 6 (IL6), and pure PBS are depicted. The difference in color depth was calculated for the B channel. Error bars indicate the standard deviation for five repeats.

The assay was tested for potential interference from other substances. Interleukin 6, butyrylcholinesterase, and human serum albumin were tested at a concentration of 50 ng/mL. None of the proteins tested provided a signal significantly different from PBS, while all the tested proteins provided a signal significantly (*p* = 0.05) lower than that observed during the assay of TNFα at 20 pg/mL. The results of the interference experiment are depicted in Figure 5. 

**Figure 6:**
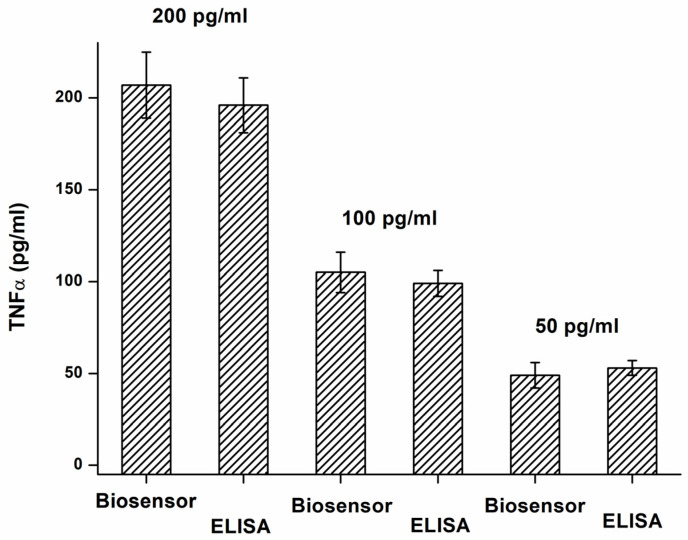
Validation of TNFα assay by biosensor (B channel) using ELISA as a standard method. Error bars indicate the standard deviation for five repeats.

The validation of the biosensor assay to the standard ELISA was another experiment that checked the plausibility of the analytical results. Both ELISA and biosensor analyzed three samples with 50, 100, and 200 pg/mL TNFα concentrations, and the results of the assays were recalculated to concentrations using calibration plots. The validation results are depicted in Figure 6. The quality of antibodies used in the assay should be considered. The low interference of the tested proteins and reliable validation tests are mainly based on the specificity of antibodies used in the assay. The biosensor assay will have the same limitations as the ELISA method. When the two methods are compared together, the biosensor assay has a lower sensitivity, but it is a substantially simpler assay. The lower sensitivity is not a significant problem because the purpose of the assay is to reveal serious pathologies in the first screening, and the level of TNFα is quite high in these pathologies. 

The effect of the matrix on the assay was another experiment that confirmed the plausibility of the results. Samples including 100 pg/mL of TNFα were prepared by solving in PBS, tap water, collected blood from healthy volunteers, human blood plasma from healthy volunteers, and urine from healthy volunteers. All samples were assayed by the standard procedure, and the change in color depth was recalculated to the concentration of TNFα using calibration in the B channel. The results of the assay are depicted in Table 1. The results did not have significant differences (ANOVA, *p* 0.05). This experiment shows that any matrix effect can be anticipated when standard clinical samples are processed. The result is substantially based on the quality of used antibodies, and the arrangement of the test. 

The biosensor presented here and its use in conditions outside laboratories complies with the common trends to prepare analytical tools for more available diagnoses. It also falls into the current trends discussed in the cited papers [33,34,35,36,37,38,39]. Despite overall simplicity, the biosensor cannot be considered a typical point-of-care device because manipulation of microplates and reagent applications has some requirements, and it is a significant simplification of laboratory equipment where standard immunoassays are used. A coupling of four webcams and appropriate low-cost hardware, such as single-board computer platforms, should be further considered if designing a similar instrument. It would thus still allow the simple introduction of an assay like the ELISA in conditions where ELISA readers are unavailable, or funds for laboratory equipment are restricted. 

The analytical specification of the biosensor assay is not better than the standard ELISA kits available on the market and has limits of detection even under pg/mL. Nevertheless, all standard ELISA kits require the use of microplate readers. The purchase price of such instrumentation typically exceeds thousands of USD/EUR. The proposed biosensor platform has a purchase price of less than approximately USD/EUR 10 for a 3D-printed platform with the light source plus the smartphone’s cost. Because any smartphone can be used, there is no need to use a specific one, and a universal device in the workplace or a private smartphone can serve for coloration recording. 

Some analytical devices based on the ELISA principle were introduced based on 3D-printed platforms [27,28,29,30]. These devices were quite effective replacements for microplate reader assays, but they still required technologies that were not easy to reproduce onsite. Smartphone-based immunoassays were also introduced and successfully tested with promising results, but with technology that was still not ready to use [40,41]. Similar results to those described here were achieved by Berg and coworkers, who decided to analyze specific antibodies against the herpes simplex virus by an analytical device based on a smartphone and 3D chassis [42]. This approach confirms that the idea of a 3D-printed device for the ELISA is feasible, though the cited study focused on antibodies of IgG isotypes occurring in high concentrations, making such an assay less difficult. 

## 4. Conclusions

Simplified devices that provide instrumental diagnostics of various pathologies and can be placed close to patients are highly desirable. They can provide faster turnaround times, reduce the distance that the biological samples should be transported or the patient should travel, save costs, and generally make medical services more accessible. This issue is not so imminent in countries with developed health services. However, such simple devices are required at sites where a mass casualty event happens, health services are not available, in healthcare provided by militaries during peacekeeping operations, etc. Only a limited number of such devices are currently available for such purposes. 

The developed colorimetric biosensor represents a simple and reliable analytical device. The combination of a common smartphone camera and 3D-printed parts makes the biosensor device highly available, and allows it to be used in improvised laboratories, poorly equipped laboratories of field hospitals, and similar sites.

Although the biosensor has been described as an analytical tool for an assay of TNFα, the biosensor platform is universal, and may be easily adapted for other analytes by replacing the specific antibodies with others. TNFα served as a model analyte, and the principle of the assay does not differ from the assay of other analytes with similar physico-chemical properties. The results of this study provide for the development of simple analytical devices easily reproducible by 3D printing and can be achieved using generally available electronics. The overall idea of the construction came from the approach of do-it-yourself analytical devices. All the experiments conducted in this work confirmed the possibility of making an analytical device capable of detecting even markers occurring in low levels, and suitable for processing common biological samples taken for examination in laboratories of clinical biochemistry and clinical immunology. 

## Figures and Tables

**Table 1:** Testing of matrix effect on assay by the colorimetric biosensor in the B channel.

Sample	TNFα (pg/mL)	RSD (%)	Recovery (%)
PBS	105 ± 11	11	-
Blood	113 ± 15	13	108
Plasma	108 ± 13	12	103
Urine	98 ± 16	16	93.3

## Data Availability

All data are included in the paper.

## References

[1] Ahirwar R., Bhattacharya A., Kumar S. (2022). Unveiling the underpinnings of various non-conventional ELISA variants: A review article. Expert Rev. Mol. Diagn..

[2] Daurat G. (2008). Yes, we should keep ABO agglutination test within bedside transfusion checks. Transfus. Clin. Biol..

[3] Björkman C., Uggla A. (1999). Serological diagnosis of Neospora caninum infection. Int. J. Parasit..

[4] Dey M.K., Iftesum M., Devireddy R., Gartia M.R. (2023). New technologies and reagents in lateral flow assay (LFA) designs for enhancing accuracy and sensitivity. Anal. Methods.

[5] Ince B., Uludag I., Demirbakan B., Özyurt C., Özcan B., Sezgintürk M.K. (2023). Lateral flow assays for food analyses: Food contaminants, allergens, toxins, and beyond. TrAC-Trends Anal. Chem..

[6] Silva G.B.L., Campos F.V., Guimaraes M.C.C., Oliveira J.P. (2023). Recent Developments in Lateral Flow Assays for Salmonella Detection in Food Products: A Review. Pathogens.

[7] Cavalier E. (2023). Determination of parathyroid hormone: From radioimmunoassay to LCMS/MS. Clin. Chem. Lab. Med..

[8] Liu R., Zhang S.X., Wei C., Xing Z., Zhang S.C., Zhang X.R. (2016). Metal Stable Isotope Tagging: Renaissance of Radioimmunoassay for Multiplex and Absolute Quantification of Biomolecules. Acc. Chem. Res..

[9] Guo M., Chen Y., Mo X.H., Wei H., Li Y.Y., Jia Y.J., Hu F.D., Du Y.L. (2024). Review-Electrochemical Immunosensors for Depression Markers Detection: Development in Recent Years. J. Electrochem. Soc..

[10] Mehta D., Gupta D., Kafle A., Kaur S., Nagaiah T.C. (2023). Advances and Challenges in Nanomaterial-Based Electrochemical Immunosensors for Small Cell Lung Cancer Biomarker Neuron-Specific Enolase. ACS Omega.

[11] Evtugyn G., Hianik T. (2019). Electrochemical Immuno- and Aptasensors for Mycotoxin Determination. Chemosensors.

[12] Bergua J.F., Alvarez-Diduk R., Idili A., Parolo C., Maymo M., Hu L., Merkoci A. (2022). Low-Cost, User-Friendly, All-Integrated Smartphone-Based Microplate Reader for Optical-Based Biological and Chemical Analyses. Anal. Chem..

[13] Volpe C., Vadstein O., Andersen G., Andersen T. (2021). Nanocosm: A well plate photobioreactor for environmental and biotechnological studies. Lab Chip.

[14] Bagheri N., Cinti S., Caratelli V., Massoud R., Saraji M., Moscone D., Arduini F. (2019). A 96-well wax printed Prussian Blue paper for the visual determination of cholinesterase activity in human serum. Biosens. Bioelectron..

[15] Seddaoui N., Amine A. (2021). Smartphone-based competitive immunoassay for quantitative on-site detection of meat adulteration. Talanta.

[16] Wang X., Wang H.Y., Wan X.Y., Li M.J., Tang D.P. (2023). Smartphone-based photoelectrochemical immunoassay for carcinoembryonic antigen based on BiOCl/CuBi2O4 heterojunction. Anal. Chim. Acta.

[17] Li H., Ying Y., Cao Z., Liu G.Y., Wang J. (2022). Research Progress on Rapid Detection Technology Based on Smartphone and Lateral Flow Immunoassay. Anal. Chim. Acta.

[18] Ben-Baruch A. (2022). Tumor Necrosis Factor α: Taking a Personalized Road in Cancer Therapy. Front. Immunol..

[19] Mikail M., Wilson A. (2021). Low Serum Tumor Necrosis Factor-α Antagonist Concentrations in Patients With Inflammatory Bowel Disease Who Achieve Healing From Pyoderma Gangrenosum. Inflamm. Bowel Dis..

[20] Cahn R.T., Zinn Z., Kolodney M.S. (2023). Tumor necrosis factor inhibitors and methotrexate are associated with decreased COVID-19-related hospitalization: Follow up of “Clinical outcomes of COVID-19 in patients taking tumor necrosis factor inhibitors and methotrexate”. J. Am. Acad. Dermatol..

[21] Vercellini P., Debenedetti F., Rossi E., Colombo A., Trespidi L., Crosignani P.G. (1993). Tumor necrosis factor in plasma and peritoneal fluid of women with and without endometriosis. Gynecol. Obstet. Investig..

[22] Cascio A., Gervasi F., Giordano S., Palazzolo B., Salsa L. (1997). Plasma levels of tumor necrosis factor-alpha and interferon-gamma in Sicilian children with Mediterranean spotted fever. Int. J. Clin. Lab. Res..

[23] Keane H.M., Sheron N., Goka J., Hughes R.D., Williams R. (1996). Plasma inhibitory activity against tumour necrosis factor in fulminant hepatic failure. Clin. Sci..

[24] Ebrahimi M., Norouzi P., Davami F., Bonakdar A., Marzabad M.A., Tabaei O. (2022). Direct detection of TNF-α by copper benzene tricarboxylate MOFs/gold nanoparticles modified electrochemical label-free immunosensor using FFT admittance voltammetry. J. Electroanal. Chem..

[25] Bari S.M.I., Reis L.G., Nestorova G.G. (2019). Calorimetric sandwich-type immunosensor for quantification of TNF-α. Biosens. Bioelectron..

[26] Yola M.L., Atar N. (2021). Novel voltammetric tumor necrosis factor-alpha (TNF-alpha) immunosensor based on gold nanoparticles involved in thiol-functionalized multi-walled carbon nanotubes and bimetallic Ni/Cu-MOFs. Anal. Bioanal. Chem..

[27] Parandakh A., Ymbern O., Jogia W., Renault J., Ng A., Juncker D. (2023). 3D-printed capillaric ELISA-on-a-chip with aliquoting. Lab Chip.

[28] Singh H., Shimojima M., Fukushi S., Van A.L., Sugamata M., Yang M. (2015). Increased sensitivity of 3D-Well enzyme-linked immunosorbent assay (ELISA) for infectious disease detection using 3D-printing fabrication technology. Bio-Med. Mater. Eng..

[29] Singh H., Shimojima M., Shiratori T., Van An L., Sugamata M., Yang M. (2015). Application of 3D Printing Technology in Increasing the Diagnostic Performance of Enzyme-Linked Immunosorbent Assay (ELISA) for Infectious Diseases. Sensors.

[30] Bauer M., Kulinsky L. (2018). Fabrication of a Lab-on-Chip Device Using Material Extrusion (3D Printing) and Demonstration via Malaria-Ab ELISA. Micromachines.

[31] Damas P., Reuter A., Gysen P., Demonty J., Lamy M., Franchimont P. (1989). Tumor necrosis factor and interleukin-1 serum levels during severe sepsis in humans. Crit. Care Med..

[32] Ferrajoli A., Keating M.J., Manshouri T., Giles F.J., Dey A., Estrov Z., Koller C.A., Kurzrock R., Thomas D.A., Faderl S. (2002). The clinical significance of tumor necrosis factor-alpha plasma level in patients having chronic lymphocytic leukemia. Blood.

[33] Xu D.D., Huang X.W., Guo J.H., Ma X. (2018). Automatic smartphone-based microfluidic biosensor system at the point of care. Biosens. Bioelectron..

[34] Omidfar K., Ahmadi A., Syedmoradi L., Khoshfetrat S.M., Larijani B. (2020). Point-of-care biosensors in medicine: A brief overview of our achievements in this field based on the conducted research in EMRI (endocrinology and metabolism research Institute of Tehran University of medical sciences) over the past fourteen years. J. Diabetes Metab. Disord..

[35] Poschenrieder A., Thaler M., Junker R., Luppa P.B. (2019). Recent advances in immunodiagnostics based on biosensor technologies-from central laboratory to the point of care. Anal. Bioanal. Chem..

[36] Kulkarni M.B., Ayachit N.H., Aminabhavi T.M. (2022). Biosensors and Microfluidic Biosensors: From Fabrication to Application. Biosensors.

[37] Hwang C., Lee W.J., Kim S.D., Park S., Kim J.H. (2022). Recent Advances in Biosensor Technologies for Point-of-Care Urinalysis. Biosensors.

[38] Pohanka M. (2024). Current trends in digital camera-based bioassays for point-of-care tests. Clin. Chim. Acta.

[39] Syed S., Rahaman A., Mondal A., Shaligram S., Pawar S.P. (2024). Diagnosis of infectious diseases: Complexity to convenience. Sens. Diagn..

[40] Taron W., Phooplub K., Sanchimplee S., Piyanamvanich K., Jamnongkan W., Techasen A., Phetcharaburanin J., Klanrit P., Namwat N., Khuntikeo N. (2021). Smartphone-based fluorescent ELISA with simple fluorescent enhancement strategy for Opisthorchis viverrini (Ov) antigen detection in urine samples. Sens. Actuator B Chem..

[41] Wang C., Wu Z., Liu B.C., Zhang P.L., Lu J.H., Li J.F., Zou P., Li T.T., Fu Y.S., Chen R.A. (2021). Track-etched membrane microplate and smartphone immunosensing for SARS-CoV-2 neutralizing antibody. Biosens. Bioelectron..

[42] Berg B., Cortazar B., Tseng D., Ozkan H., Feng S., Wei Q.S., Chan R.Y.L., Burbano J., Farooqui Q., Lewinski M. (2015). Cellphone-Based Hand-Held Microplate Reader for Point-of-Care Testing of Enzyme-Linked Immunosorbent Assays. ACS Nano.

